# Childhood adversity, allostatic load and epigenetic signatures in paediatric and adult-onset multiple sclerosis

**DOI:** 10.1093/braincomms/fcaf512

**Published:** 2026-01-03

**Authors:** Kimberly A O’Neill, Bernard K van der Veer, Leigh Charvet, Nadine Azmy, Steven Friedman, Jiyuan Hu, Kevin Lei, Robin Ortiz, Shayna Pehel, Yidan Shi, Anna Sosa, Kian Peng Koh, Mirjana Maletic-Savatic, Lauren B Krupp

**Affiliations:** Department of Neurology, NYU Grossman School of Medicine, New York, NY 10016, USA; Department of Development and Regeneration, KU Leuven, Leuven 3000, Belgium; Department of Neurology, NYU Grossman School of Medicine, New York, NY 10016, USA; Department of Neurology, NYU Grossman School of Medicine, New York, NY 10016, USA; Department of Population Health, NYU Grossman School of Medicine, New York, NY 10016, USA; Department of Population Health, NYU Grossman School of Medicine, New York, NY 10016, USA; Department of Molecular and Cellular Biology, Baylor School of Medicine, Houston, TX 77030, USA; Department of Population Health, NYU Grossman School of Medicine, New York, NY 10016, USA; Department of Neurology, NYU Grossman School of Medicine, New York, NY 10016, USA; Department of Population Health, NYU Grossman School of Medicine, New York, NY 10016, USA; Department of Neurology, NYU Grossman School of Medicine, New York, NY 10016, USA; Department of Development and Regeneration, KU Leuven, Leuven 3000, Belgium; Department of Molecular and Cellular Biology, Baylor School of Medicine, Houston, TX 77030, USA; Department of Molecular and Cellular Biology, Baylor School of Medicine, Houston, TX 77030, USA; Department of Neurology, NYU Grossman School of Medicine, New York, NY 10016, USA

**Keywords:** paediatric multiple sclerosis, childhood adversity, allostatic load, epigenetics

## Abstract

Childhood adversity is increasingly recognized as a critical modifier of neurologic disorder development and disease severity, including in the neuroimmune disorder multiple sclerosis (MS). While previous studies have linked early-life adversity to increased MS susceptibility and more severe disease, the underlying biological mechanisms remain poorly understood. This study investigated associations between childhood adversity and MS clinical features, with a focus on two potential pathogenic mechanisms: allostatic load and epigenetic modifications. We evaluated 60 consecutively enrolled young adults with MS; 30 with paediatric-onset MS (POMS) and 30 with adult-onset MS (AOMS). At time of enrolment in this cross-sectional study, participants had MS disease duration of 6 years on average. POMS participants were mean 22.09 (2.66) years and AOMS participants were mean 32.41 (2.19) years old. 62% of participants were female. Childhood adversity was defined using a composite index of individual, family and socioeconomic measures captured by the adverse childhood experiences questionnaire, parental education level and estimated household income during childhood. Clinical outcomes included patient-reported SymptoMScreen questionnaire regarding MS symptom burden and MS neurologist-assessed disability using the Expanded Disability Status Scale (EDSS) of the participant’s neurologic exam at the time of enrolment. Circulating biomarkers of allostatic load and genome-wide epigenetic profiles (DNA methylation via RRBS; reduced representation bisulfite sequencing) were also assessed. A history of high childhood adversity was associated with significantly greater patient-reported MS symptom burden (*P* = 0.001) and higher neurologist-reported EDSS disability scores (*P* = 0.028), independent of disease duration or timing of treatment initiation. There were no differences between childhood adversity and circulating biomarkers of allostatic load. While childhood adversity was not associated with global epigenetic changes across the entire cohort, stratified analysis revealed divergent methylation patterns by age of MS onset: POMS participants with childhood adversity had increased DNA methylation, whereas AOMS participants with childhood adversity showed decreased methylation compared to individuals without childhood adversity. None of the observed clinical and biologic differences were explained by differences in disease duration or the interval between symptom onset and treatment initiation. Our findings suggest that childhood adversity is associated with increased MS symptom burden and neurologic disability in young adults with MS. Childhood adversity may differentially shape the epigenome, depending on the age of MS onset, with potential implications for disease trajectory and therapeutic vulnerability. These results support the biological embedding of childhood adversity in MS and highlight the need for age- and exposure-sensitive approaches to understanding MS pathogenesis across the lifespan.

## Teaser

O’Neill *et al*. have studied 60 young adults, 30 with paediatric-onset multiple sclerosis (POMS) and 30 with adult-onset MS (AOMS). They found that participants exposed to childhood adversity had worse MS symptoms and greater MS disability. Individuals with POMS and childhood adversity had hypermethylated signatures on epigenetic analyses.

## Graphical Abstract

**Graphical Abstract:**
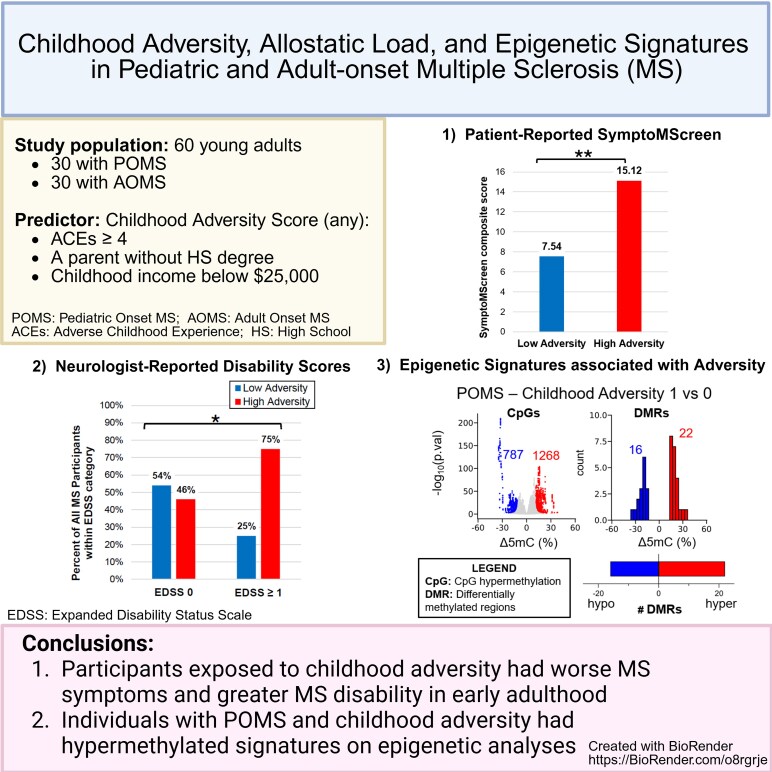

## Introduction

Multiple sclerosis (MS) is a chronic, immune-mediated disorder of the central nervous system, characterized by inflammation, demyelination and neurodegeneration. While the precise pathogenesis of MS remains elusive, it is widely accepted that a complex interplay between genetic predisposition and environmental exposures contributes to disease onset and progression. Notably, environmental exposures early in life may exert lifelong physiological effects, particularly during sensitive developmental windows.

In up to 5–10% of cases,^1^ MS manifests exceptionally early, before the age of 18 years—a subset termed paediatric-onset MS (POMS). Compared to adult-onset MS (AOMS), individuals with POMS are temporally closer to their childhood exposures and typically have fewer comorbidities,^2^ making this cohort particularly well-suited for investigating the biological consequences of early-life environmental influences.

One such early life influence is childhood adversity, potentially traumatic or chronically stressful experiences that happen early in life, which has been tied to an increased risk of chronic illnesses and earlier mortality.^3^ Childhood adversity can be measured with the adverse childhood experiences (ACEs) questionnaire but can also include broader social drivers of health such as socioeconomic status and education. Emerging research suggests that childhood adversity may not only increase the risk of MS but also affect its clinical course.^4^ A recent systematic review concluded that there is a significant association between ACEs and MS development, MS relapse frequency, and earlier MS onset.^5^ Further, in POMS cohorts, those in families experiencing socioeconomic hardship—defined as having public insurance or low parental education—demonstrate significantly earlier MS onset,^6^ while individuals with POMS who grew up in socially disadvantaged neighbourhoods had greater disease burden on brain MRI.^7^ These findings raise important questions about the biological pathways by which early adversity influences MS onset and severity, particularly in paediatric and young adult individuals.

Two proposed mechanisms through which childhood adversity may lead to chronic disease are allostatic load and epigenetic modifications.^8,9^ Allostatic load refers to the cumulative physiological wear and tear from prolonged activation of the stress response,^10,11^ measurable through serum biomarkers such as C-reactive protein, and it has been associated with accelerated ageing and reduced resiliency to disease over time.^9^ Epigenetic modifications—such as DNA methylation—represent a molecular interface through which environmental exposures alter gene expression without changing the DNA sequence. Both childhood adversity and MS have independently been tied to accelerated epigenetic ageing compared to matched controls,^12,13^ yet little is known about how these processes may interact.

Despite increasing recognition of the potential links between childhood adversity and biologic changes leading to worse outcomes later in life for chronic disorders, a major gap in the current research landscape remains:^14^ there has been no integrated investigation of social adversity, allostatic load, epigenetic mechanisms and MS clinical outcomes within a unified cohort, particularly among individuals with paediatric-onset disease. Most studies to date have examined these domains in isolation, limiting our understanding of how childhood adversity becomes biologically embedded to influence MS risk and severity. To address this critical knowledge gap, we designed a pilot study to delve deeper into the associations between childhood adversity, MS disease severity, biomarkers of allostatic load and epigenetic signatures in a cohort of young adults with MS, with either paediatric or adult onset.

We hypothesized that individuals with a history of childhood adversity would exhibit greater MS symptom burden and neurologic deficits, increased allostatic load and distinct epigenetic modifications. We additionally surmised that these associations would be more pronounced in individuals with POMS due to their proximity to early-life exposures.

## Materials and methods

### Participants

This is a cross-sectional pilot study of 60 MS participants recruited during routine clinical care in an outpatient practice at the NYU Langone Multiple Sclerosis Comprehensive Care Center in New York. A total of 30 POMS and 30 AOMS patients who met eligibility criteria were consecutively enrolled between August 2022 and March 2023. All were young adults, but recruitment was age-stratified so that patients were closely matched on disease duration. POMS participants were aged 18–25 years at time of enrolment and had MS onset before age 18 years. AOMS participants were aged 26–35 years at time of enrolment and had MS onset after age 21 years. All clinic visits were with clinicians that accepted the same health insurances. A pre-screening medical record review was performed by the study team on all MS patients who fit age range eligibility. The first three eligible patients of the day were approached for study inclusion, to improve temporal recruitment over multiple months. Eligibility criteria included a confirmed diagnosis of relapsing-remitting MS (RRMS), the ability to provide consent, and self-reported fluency in English language. Diagnosis of RRMS was made by an MS-trained neurologist according to international criteria.^15^ Patients were excluded if they had another major neurologic, psychiatric, or medical condition other than MS judged by the treating clinician to be primary (e.g. uncontrolled seizure disorder, traumatic brain injury, cancer). Written consent was obtained from all participants in accordance with the Declaration of Helsinki and the study was approved by the NYU Institutional Review Board. Participants were compensated with a $100 gift card for their time.

### Procedures

We collected general demographic information including current age, sex, current health insurance type and self-reported race and ethnicity. Information about parental education level and household income during childhood was also collected.

#### Self-report questionnaires

Participants completed self-report questionnaires including the ACEs, a widely used 10-point questionnaire of childhood adversity (abuse, neglect, household dysfunction).^3^ The number of adversity exposures is totalled to calculate a composite ACE score between 0 and 10 with higher values representing greater childhood adversity. Scores were reported by the commonly used cutoff ACEs ≥4^3,16^ as a score of ≥4 has been used as a moderate-severe level of childhood adversity.^16^ Given the sensitive nature of the ACEs questionnaire, a social worker was available for support if needed. Participants also completed the Symptom Burden self-assessment (SymptoMScreen),^17^ a 12-item questionnaire inventory in which participants rate their experience across common MS symptoms (such as fatigue, mood, cognition, and sensory symptoms). Each symptom is scored 0–6, and a composite score is calculated by totalling all questions for a range of 0–72 with higher scores representing greater symptom burden of disease.

#### Clinical evaluation

An Expanded Disability Status Score (EDSS)^18^ was completed by the patient’s neurologist at the time of enrolment based on their neurologic examination on date of enrolment. The neurologist was blinded to any patient questionnaire responses. The Symbol Digit Modalities Test,^19^ a measure of cognitive processing speed, was administered by a trained team member on date of enrolment. MS disease information was obtained via chart review including age of symptom onset and type of disease-modifying therapy (DMT). Additional medical data collected at enrolment included the body mass index (BMI), blood pressure, heart rate, and presence of any medical comorbidities.

### Allostatic load biomarkers

All participants provided research blood and urine samples collected at enrolment. Ten allostatic load biomarkers were analysed per previously published Index of Cardiometabolic Health, an allostatic load index that can be obtained from routinely collected samples.^20^ Serum biomarkers included: albumin, HgA1c, total cholesterol, high-density lipoproteins (HDL), low-density lipoproteins (LDL), triglycerides and serum creatinine. Given our interest in inflammation, we replaced eGFR with serum C-reactive protein. Urine was analysed for urine albumin/creatinine ratio.

#### Creation of reference samples

*NHANES 2017–2018.* To create a reference sample of age- and gender-matched controls to analyse the biomarkers, we utilized the NHANES dataset.^21^ The most complete dataset available at time of analysis was the 2017–2018 NHANES dataset as NHANES data collection was interrupted by the 2020 COVID-19 pandemic. Four age- and gender-matched reference samples were created: males aged 18–26 years, females aged 18–26 years, males aged 27–35 years and females aged 27–35 years. Mean values of the 10 allostatic load biomarkers were created for each subset using available NHANES data.

#### Creation of allostatic load z-scores

*MS samples.* All 60 MS patients had their 10 biomarker results compared to their age- and sex-matched normative mean, to create a z-score for each biomarker. Worse markers of poor health were represented by higher, positive z-scores. HDL and albumin levels were multiplied by −1 to represent lower numbers as representing poorer health. The average of all individual biomarker z-scores indicated their allostatic load index.^20^

### Epigenetic analysis

#### Epigenetic blood sample assessment

The initial seven patients enrolled were not consented for epigenetic testing as testing was only available later in the study. However, all subsequent 53 participants consented to their blood samples being used for epigenetic and methylation analysis. Peripheral blood mononuclear cells (PBMCs) were obtained from these 23 patients with POMS and 30 patients with AOMS. To map DNA methylation differences between patient groups, we performed reduced representation bisulfite sequencing (RRBS)^22^ on the 53 PBMC sample cohort. Libraries were prepared using Zymo-Seq RRBS™ Library Kit and were sequenced for 15–20 million read pairs per sample. The results analysed CpG hypermethylation, as CpG sites are especially important in methylation changes and gene silencing. Differentially methylated regions (DMRs), genomic regions with different DNA methylation patterns, were also analysed as they are important in gene expression regulation.^23^

#### Sequencing analysis

Raw reads were first trimmed for quality and presence of the adaptor using TrimGalore (v0.6.4_dev). Processed reads were aligned to hg38 using Bismark (v0.22.3) followed by methylation extraction using methyldackel (v0.5.0). Further downstream analysis was done using R (v4.0.5), together with the bsseq package (v1.36.0). CpGs were filtered based on the following criteria: (i) each CpG had to be covered at least once in every sample; (ii) coverage per library had to fall below the 99.9th percentile to eliminate potential PCR artifacts and (iii) within each group, a minimum of 80% of samples had to exhibit a coverage of at least 5× at the CpG site. After filtering, we retained 3.1 × 10^6^ CpGs, with a median coverage of 19X per library, covering roughly 11% of hg38. For dendrogram and PCA analysis, CpGs were retained based on the 90th percentile of standard deviation across samples, retaining the most variable CpG sites. For differential methylation analysis, the DSS package (v2.48.0) was used based on the dispersion shrinkage method followed by the Wald statistical test for beta-binomial distributions. First smoothing was applied using BSsmooth function implemented in the bsseq framework. Then, differential CpGs were called where false discovery rate (FDR) < 0.05 and ΔCpG 5 mC > 10%. From these differential CpGs, DMRs were called with FDR < 0.01. DMRs were selected with the following criteria: Δ 5 mC > 10%, n CpGs ≥ 3 and length > 100 bp. To prevent false positive DMRs based on coverage differences between conditions, we kept DMRs where the coverage of the DMR was less than 3× the genome wide average per library, and where the total coverage difference between two groups is less than 5×. All filtering steps were done on unsmoothed data. Genes were associated with CpGs using the rGREAT (v1.22.0) package.

### Conceptual framework

#### Predictors—childhood adversity score

The childhood adversity score, involving individual, family, and socioeconomic adversity, was adapted from Wilson 2024.^6^ Childhood adversity was defined as an individual who met any of the following criteria: elevated ACEs scores ≥4, a parent without a high school degree, or low childhood household income below $25 000. This income bracket cutoff was chosen as it would include the federal poverty level for a family of four in early 2000s,^24^ when most of our young adult participants were children. Insurance type during childhood was not collected and therefore insurance was not included in the score.

#### Outcomes—MS and health outcomes

**Figure 1:**
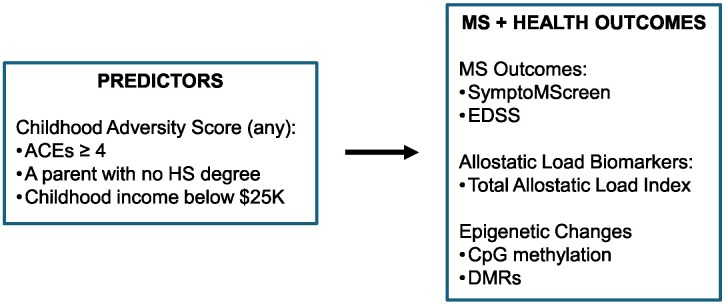
**Conceptual framework for study design**.

As shown in Fig. 1, our measures of interest were conceptualized in three parts: MS outcomes included the SymptoMScreen composite and EDSS; allostatic load index was the total composite of allostatic load biomarkers and epigenetic changes included CpG hypermethylation and DMR.

### Statistical analysis

Data were assessed for completeness, and variables with missing values were examined for patterns of missingness. As missing data were minimal, imputation methods were not applied. Descriptive statistics were used to summarize demographic and clinical characteristics. Means and standard deviations (SD) or median and interquartile range were reported for continuous variables and frequencies and percentages for categorical variables. Continuous measures were compared between groups using the two-sided independent sample *t*-tests. As EDSS scales are ordinal, non-parametric Mann–Whitney *U* tests were used. Chi-square tests or Fisher’s exact tests were used for categorical variables when appropriate. A *P*-value threshold of <0.05 was used to determine statistical significance. Statistical analyses were conducted using SPSS software (Version 28.0. Armonk, NY: IBM Corp) for demographic and clinical analyses and R software (v4.0.5) for epigenetic analyses.

Sample characteristics were first analysed by MS subgroup, POMS versus AOMS. Then, all results were analysed between individuals who met criteria for the childhood adversity score and individuals who did not meet the childhood adversity score criteria. Stratified results were also reported among the POMS subgroup and among the AOMS subgroup.

## Data availability

Data underlying this study are available from the corresponding author on reasonable request.

## Results

### Demographic and MS features of patient subgroups

POMS and AOMS subgroups had similar MS disease durations: 6.09 ± 2.90 versus 6.16 ± 3.53 years but consistent with eligibility criteria, had different ages at enrolment (Table 1). More POMS versus AOMS participants self-reported their race as Black (30.0% versus 23.3%), while the percent reporting Hispanic or Latino ethnicity was similar (33.3% versus 30.0%). The POMS versus AOMS subgroup had a lower median EDSS (0.00 versus 1.00, *P* = 0.007), although both subgroups had relatively little neurologic impairment. POMS participants had shorter time from MS symptom onset to diagnosis and MS symptom onset to DMT start, compared to AOMS. POMS participants were also more likely to have a history of/current high efficacy DMT use. Additional current sociodemographic features are shown in Supplementary Table 1.

**Table 1:** Features for POMS and AOMS groups

	Paediatric-onset MS (*n* = 30)	Adult-onset MS (*n* = 30)	*P*-value
Demographic features^a^			
Age at study enrolment	22.09 (2.66)	32.41 (2.19)	***<0***.***001***
*Sex* (n, %)			0.791
Male	11 (36.7%)	12 (40.0%)	
Female	19 (63.3%)	18 (60.0%)	
*Race*			0.390
White	13 (43.3%)	17 (56.7%)	
Black	9 (30.0%)	7 (23.3%)	
American Indian or Alaskan	0 (0%)	0 (0%)	
Asian	1 (3.3%)	2 (6.7%)	
Native Hawaiian or Pacific Islander	0 (0%)	0 (0%)	
Other	4 (13.3%)	4 (13.3%)	
Unknown	3 (10.0%)	0 (0%)	
*Ethnicity*			0.961
Hispanic/Latino	10 (33.3%)	9 (30.0%)	
Not Hispanic/Latino	18 (60.0%)	19 (63.3%)	
Other	2 (6.7%)	2 (6.7%)	
Age of MS onset	16.01 (1.24)	26.24 (3.18)	***<0***.***001***
Disease duration at time of enrolment	6.09 (2.90)	6.16 (3.53)	0.925
Time from MS symptom onset to MS diagnosis	0.22 (0.28)	0.97 (1.81)	***0***.***032***
Time from MS symptom onset to first DMT	0.66 (0.90)	1.42 (1.86)	***0***.***051***
*Ever on high-efficacy DMT*			***0***.***044***
Current/history of high-efficacy DMT	29 (96.7%)	24 (80.0%)	
Never on high efficacy DMT	1 (3.3%)	6 (20.0%)	
*Current DMT by efficacy*			0.095
High efficacy	27 (90.0%)	22 (73.3%)	
Moderate efficacy or none	3 (10.0%)	8 (26.7%)	
EDSS, median [IQR]	0.00 [0.00–0.25]	1.00 [0.00–2.25]	***0***.***007***
SDMT z-score, mean (SD)	−0.19 (1.38)	−0.68 (1.43)	0.182
**Childhood adversity factors**			
*Participant ACE score*			0.222
Composite ACE < 4	25 (83.3%)	21 (70.0%)	
Composite ACE ≥ 4	5 (16.7%)	9 (30.0%)	
*Mother education*			0.386
Some high school	4 (13.3%)	2 (6.7%)	
High school or GED	8 (26.7%)	6 (20.0%)	
Some college	4 (13.3%)	5 (16.7%)	
Associate’s degree or trade school	0 (0.0%)	3 (10.0%)	
Bachelor’s degree	7 (23.3%)	6 (20.0%)	
Graduate degree	4 (13.3%)	4 (13.3%)	
Missing/unknown	3 (10.0%)	4 (13.3%)	
*Father education*			0.488
Some high school	3 (10.0%)	2 (6.6%)	
High school or GED	5 (16.7%)	5 (16.7%)	
Some college	9 (30%)	2 (6.7%)	
Associate’s degree or trade school	2 (6.6%)	1 (3.3%)	
Bachelor’s degree	4 (13.3%)	9 (30.0%)	
Graduate degree	5 (16.7%)	5 (16.7%)	
Missing/unknown	2 (6.7%)	6 (20.0%)	
*Average household income during childhood*			0.376
<$25 000	3 (10.0%)	2 (6.7%)	
$25 000–$49 999	4 (13.3%)	1 (3.3%)	
$50 000–$74 999	2 (6.7%)	5 (16.7%)	
$75 000–$99 999	5 (16.7%)	2 (6.7%)	
>$100 000	5 (16.7%)	7 (23.3%)	
Unknown	11 (36.7%)	9 (30.0%)	
Missing	0 (0%)	4 (13.3%)	

^a^Age, disease duration, time reported in mean years (SD) and *P*-values are reported from independent *t*-tests. EDSS reported in median [IQR] and *P*-values are reported from Mann–Whitney *U* test. *P* values < 0.05 are shown in bold italics.

### Childhood adversity factors among POMS and AOMS subgroups

There were no significant differences between the POMS and AOMS subgroups with respect to the ACEs score of ≥ 4 as shown in Table 1. No group differences in childhood household income levels were observed, but over 40% of participants did not know their average household income during childhood.

### POMS and AOMS clinical features (Supplementary Table 2)

As shown in the supplement, the POMS and AOMS groups had similar SymptoMScreen scores except for more bladder control symptoms in the AOMS subgroup (1.167) compared to the POMS subgroup (0.500), *P* = 0.040. There were no significant differences between POMS versus AOMS in total SymptoMScreen scores, 11.13 (7.75) versus 12.53 (12.00). *P* = 0.593, respectively. There was a significant difference among EDSS scores between POMS and AOMS subgroups with median EDSS 0.00 [0.00–0.25] for the POMS subgroup and median EDSS 1.00 [0.00–2.25] for the AOMS subgroup, *P* = 0.007. There were no significant differences in the allostatic load composite index between POMS versus AOMS, 0.181 versus −0.024, *P* = 0.068, although there were significantly higher total cholesterol values and LDL values for the POMS subgroup compared to the AOMS subgroup. Both MS subgroups had negative BMI z-scores, representing lower BMI’s than the age- and sex- matched NHANES controls, as well as negative HgbA1c values. There were no significant demographic differences when the cohort was compared between patients meeting criteria for high childhood adversity and individuals with low childhood adversity (Supplementary Table 3).

### History of childhood adversity and MS outcomes

#### Childhood adversity and SymptoMScreen

As shown in Table 2, childhood adversity was significantly associated with a greater SymptoMScreen composite score for all participants (7.54 versus 15.12, two-sided independent *t*-test *t* = −3.35, *P* = 0.001). In analyses stratified by MS subgroup, the AOMS participants showed greater SymptoMScreen among individuals with high childhood adversity, while POMS participants only showed a trend.

**Table 2:** Childhood adversity and the relationship to SymptoMScreen composite

SymptoMScreen Mean (SD)	Childhood adversity, low	Childhood adversity, high	*P*-value	Mean difference (95% CI)
All Participants	*n* = 26 7.54 (5.93)	*n* = 34 15.12 (11.31)	***0***.***001***	−7.58 (−12.12 to −3.04)
POMS	*n* = 14 8.50 (5.83)	*n* = 16 13.44 (8.63)	0.081	−4.94 (−10.53 to 0.65)
AOMS	*n* = 12 6.42 (6.10)	*n* = 18 16.61 (13.32)	***0***.***020***	−10.19 (−18.64 to −1.75)

SymptoMScreen composite score. Childhood adversity was defined as meeting any of the following: ACEs ≥ 4, low parent education, and low household income during childhood. Significance of independ*e*nt sample *t*-tests and mean difference (95% confidence interval) are shown. *P* values < 0.05 are shown in bold italics.

#### Childhood adversity and EDSS scores

**Figure 2:**
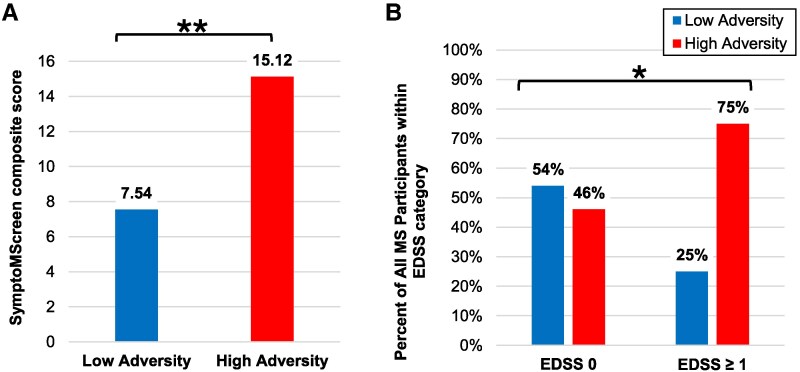
**Clinical MS outcomes by history of childhood adversity.** Results among all MS participants. Legend: *P < 0.05 and **P < 0.01 (**A) SymptoMScreen Composite scores** were greater among participants with high childhood adversity (*n* = 34, mean 15.12) compared to low childhood adversity (*n* = 26, mean 7.54) with two-sided independent *t*-test *t* = −3.35, *P* = 0.001). **(B) EDSS scores** were greater among high childhood adversity participants compared to individuals who experienced low childhood adversity. Among individuals with EDSS scores above 0, 6/24 (25%) had history of low childhood adversity compared to 18/24 (75%) with history of high childhood adversity, Fisher’s exact test, *P* = 0.034. Percents of participants within each adversity group by EDSS of 0 and EDSS ≥1 are shown.

As shown in Table 3, childhood adversity was significantly associated with higher (worse) EDSS scores for all participants (0.0 versus 1.0, Mann–Whitney *U* = 298.500, *P* = 0.028). In analyses stratified by MS subgroup, the POMS participants showed higher EDSS scores among individuals with high childhood adversity (Mann–Whitney *U* = 63.000, *P* = 0.043), while AOMS participants did not (Mann–Whitney *U* = 88.500, *P* = 0.642). A comparison of EDSS scores above 0 is shown in Fig. 2 with the raw data shown in Supplementary Table 4. Among individuals with EDSS scores above 0, 6/24 (25%) had history of low childhood adversity compared to 18/24 (75%) with history of high childhood adversity, Fisher’s exact test with odds ratio 3.56, *P* = 0.034.

**Table 3:** Childhood adversity and the relationship to EDSS scores

EDSS Median [IQR]	Childhood adversity, low	Childhood adversity, high	*P*-value	Mean Difference (95% CI)
All Participants	*n* = 25 0.00 [0.00–0.05]	*n* = 34 1.00 [0.00–2.00]	***0***.***028***	−0.58 (−1.14 to −0.02)
POMS	*n* = 14 0.00 [0.00–0.00]	*n* = 16 0.00 [0.00–1.75]	***0***.***043***	−0.75 (−1.28 to −0.22)
AOMS	*n* = 11 1.00 [0.00–2.00]	*n* = 18 1.00 [0.00–2.50]	0.642	−0.27 (−1.25 to 0.72)

Childhood adversity was defined as meeting any of the following: ACEs ≥ 4, low parent edu*c*ation, and low household income during childhood. Significance of Mann–Whitney *U*-test and mean difference (95% confidence interval) are shown. *P* values < 0.05 are shown in bold italics.

### History of childhood adversity and allostatic load biomarkers

#### Childhood adversity and allostatic load composite

As shown in Table 4, childhood adversity was not associated with greater allostatic load composite scores for all participants or when analyses were stratified by POMS versus AOMS subgroups.

**Table 4:** Childhood adversity and the relation to allostatic load composite z-scores

AL z-scores Mean (SD)	Childhood adversity, low	Childhood adversity, high	*P*-value	Mean difference (95% CI)
All Participants	*n* = 26 0.04 (0.46)	*n* = 34 0.11 (0.42)	0.515	−0.07 (−0.30 to 0.15)
POMS	*n* = 14 0.13 (0.53)	*n* = 16 0.22 (0.32)	0.552	−0.09 (−0.42 to 0.23)
AOMS	*n* = 12 −0.07 (0.34)	*n* = 18 0.01 (0.48)	0.611	−0.08 (−0.41 to 0.25)

Childhood adversity was defined as meeting any of the following: ACEs ≥ 4, low parent e*d*ucation, and low household income during childhood.

Composite AL z-scores were compared between individuals with low childhood adversity versus high childhood adversity, first among all participants and then stratified by POMS and AOMS subgroups. Significance of independent sample *t*-tests and mean difference (95% confidence interval) are shown.

### History of childhood adversity and epigenetic changes

#### Initial data exploration

Hierarchical clustering plot and principal component analysis of CpG methylation are shown in the supplement (Supplementary Fig. 1). No segregation was discernible based on patient subtypes and history of childhood adversity, suggesting that methylomes are globally similar in the entire cohort.

#### Comparisons among all participants

In an analysis of the entire cohort of MS participants, we did not find any statistically significant differentially methylated CpGs or DMRs when comparing high versus low childhood adversity.

#### Comparisons across MS subgroups

**Figure 3:**
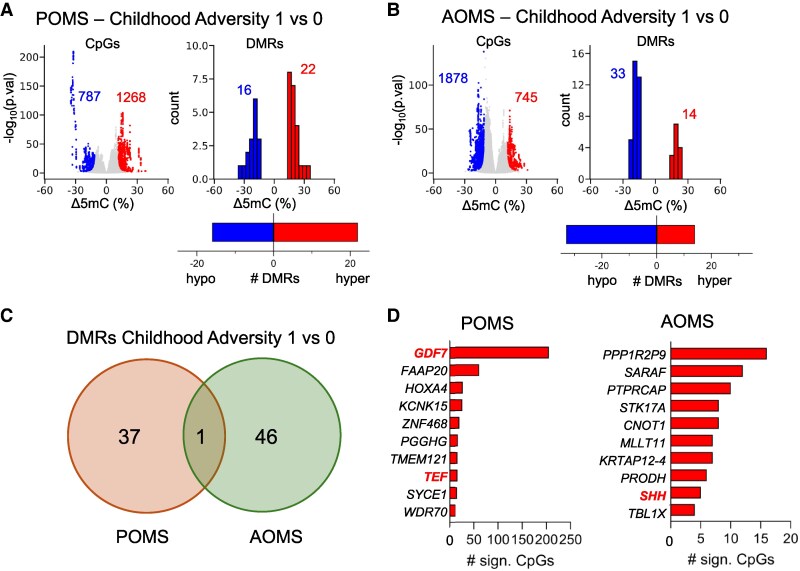
**Epigenetic signatures of childhood adversity among POMS and AOMS subgroups.** DNA methylation changes associated with childhood adversity among POMS (*n* = 30) versus AOMS (*n* = 30) subgroups. (**A, B)**. Volcano plots of differentially methylated CpGs (left) and frequency histograms of differentially methylated regions (right) classified by increased (hyper, red) or decreased (hypo, blue) methylation associated with a history of childhood adversity in paediatric (**A**) and adult (**B**) onset MS groups. X-axis measures methylation differences, binned in 10% intervals in the DMR histogram. Differentially methylated CpGs were called where FDR < 0.05 and ΔCpG 5 mC > 10%, from which DMRs were called with FDR < 0.01, based on the Wald test for beta-binomial distributions in the DSS package. 1 versus 0, history of high childhood adversity versus low. (**C)** Venn overlap of DMRs in the two patient groups. (**D)** Top 10 genes with TSS within 5 kb of hypermethylated CpGs, associated with ACEs in AOMS and POMS groups. Total number (n) of genes per comparison group is indicated within parentheses. Genes specifically associated with neuroinflammation and/or neurodegeneration are highlighted in red.

When stratified by MS subgroup, we observed striking differences associated with childhood adversity in both the POMS and AOMS subgroups (Fig. 3). Specifically, we identified 2055 significantly differentially methylated CpGs in the POMS subgroup and 2623 in the AOMS subgroup. These CpGs in the POMS subgroup corresponded to 38 DMRs associated with childhood adversity, while in the AOMS subgroup, there were 47 DMRs (Fig. 3A, B). Remarkably, in the POMS subgroup, 60% of significant CpGs gained methylation (hyper-methylation), while in the AOMS subgroup, ∼70% exhibited loss of methylation (hypo-methylation). Adversity-associated DMRs in the two groups show little overlap (Fig. 3C). Thus, childhood adversity can be associated with changes in DNA methylation, but the effects are distinct in different patient subtypes classified by age of disease onset. The prevalence of hyperDMRs in the adversity-exposed POMS group suggests that a hypermethylation signature may capture adversity exposure associated with early onset of MS.

#### Genes with differentially methylated regions

**Figure 4:**
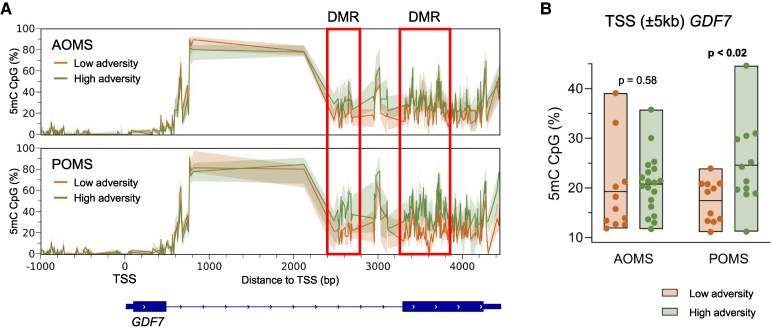
**Epigenetic changes for GDF7 gene in POMS and AOMS. Hypermethylated regions in *GDF7* associated with childhood adversity in POMS.** (**A**) Median CpG methylation levels plotted per CpG over the *GDF7* gene locus. The hyper DMR containing 205 significant CpGs identified from differential analysis of POMS patient subsets exposed to ‘high childhood adversity’ versus ‘low childhood adversity’ are boxed in red. Green and orange shades denote interquartile range in ‘high childhood adversity’ exposed versus ‘low childhood adversity’ respectively. (**B**) Box plots of methylation levels of the entire GDF7 gene locus (± 5 kb from TSS) in patients stratified by MS subgroup and childhood adversity history. *P*-values for both AOMS and POMS were obtained using two-sided independent *t*-tests, followed by multiple-testing correction using the Benjamini, Krieger, and Yekutieli procedure. Each dot represents mean CpG methylation levels per patient. The box corresponds to the interquartile range and the middle line is the median. The cohort included 23 POMS participants (11 low adversity, 12 high adversity) and 30 AOMS participants (11 low adversity, 19 high adversity).

Because DMRs captured by RRBS were enriched at promoter regions, we used the Genomic Regions Enrichment of Annotations Tool (GREAT) to identify genes with transcription start sites residing within 5 kb of hypermethylated CpGs (Fig. 3D). We detected about twice as many genes with adversity-associated hyperDMRs in POMS than in AOMS. Interestingly, the top DMR in the paediatric group, associated with 205 significantly differentially methylated CpGs, was found at the transcription start sites (TSS) of *GDF7*, which encodes a secreted ligand of the TGFβ superfamily of proteins involved in neuroinflammation.^25^ These 205 CpGs were concentrated within two DMRs in intron 1 and exon 2, respectively 240–260 bp and 320–390 bp downstream of the TSS, showing significantly higher methylation levels in POMS patients classified with high adversity compared to those without, but no difference in AOMS patients (Fig. 4).

## Discussion

In this study, we investigated the relations between childhood adversity, MS clinical outcomes, allostatic load, and epigenetic signatures in young adult individuals with POMS and AOMS. Across the full cohort of 60 individuals, childhood adversity was significantly associated with greater MS symptom burden and greater neurologic disability, even at this early stage of disease. Furthermore, we identified specific epigenetic signatures associated with childhood adversity that differ by age of MS onset.

Among all participants—and particularly in the AOMS subgroup—childhood adversity was associated with significantly higher SymptoMScreen scores, indicating worse patient-reported MS symptom severity. Specific domains, including walking, gait, pain, fatigue, vision, cognitive function, and depression, and the overall composite scores were all significantly more severe among individuals that experienced high childhood adversity compared to those with low childhood adversity. These findings are consistent with prior work linking ACEs and socioeconomic disadvantage to greater neurologic symptomatology and reduced quality of life in MS.^4,26-28^ The very low symptom burden among the POMS group may potentially contribute to the lack of an association.

Neurologist-assessed disability corroborated these findings—participants with childhood adversity had significantly higher EDSS scores, a trend most apparent in the POMS subgroup. Notably, EDSS scores were low across all groups, consistent with the minimal neurologic impairment typical for this population with relatively short disease duration.^29^ One interpretation of the association of childhood adversity with greater neurologic impairment is that early adversity subtly compromises an individual’s resiliency to chronic disease. It is plausible that individuals with a history of childhood adversity experience greater difficulty recovering from attacks, potentially leading to cumulative neurologic impairment over time. This pattern aligns with the theory that childhood adversity creates a ‘toxic stress response’, where chronic psychosocial stress accelerates physiologic ageing and reduces reserve across multiple systems.^30^

Contrary to our hypothesis that childhood adversity would be linked with elevated biomarkers of allostatic load, such as CRP or lipid indices, no association was found. A 2022 study of the allostatic load index in 90 patients with relapsing-remitting MS in Poland found individuals with MS had greater allostatic load indices compared to healthy controls. MS patients in this Polish cohort had mean age of 42 years.^31^ The results of our pilot study may suggest that our patients were too young to see the full effects of allostatic load, which accumulates over time, and perhaps requires older ages to be detected.

A striking finding was the identification of distinct DNA methylation patterns associated with childhood adversity, particularly among POMS participants. In this group, adversity was associated with widespread hypermethylation. Hypermethylation is an epigenetic finding known to be particularly biologically relevant.^32^ It is noteworthy that hypermethylation was specifically observed in *GDF7*, a gene encoding a TGF-β family ligand involved in neuroinflammation and neural development.^25^ Hypermethylation in *GDF7* may reflect gene silencing with downstream implications for immune regulation and neuronal resilience—mechanisms that could plausibly mediate the effect of early adversity on disease expression. In contrast, AOMS participants with childhood adversity exhibited a different epigenetic pattern—hypomethylation across various regions—highlighting the potential importance of developmental timing in shaping epigenetic responses. Identification of *GDF7* as a candidate gene linking adversity to MS pathology warrants further functional investigation. The divergent epigenetic signatures between POMS and AOMS suggest that the biological consequences of adversity could be age-dependent, with early-life adversity leaving a more pronounced and possibly more pathogenic epigenetic imprint among those with a younger onset of MS.

Overall, the finding linking childhood adversity and hypermethylation in those with POMS and hypomethylation among those with AOMS adds to a growing body of literature positioning the epigenome as a critical interface between environmental exposures and chronic disease risk.^32,33^ Childhood appears to be an especially sensitive time to experience adversity, as it can alter the HPA axis that plays a critical role in regulating stress response, mood, immune function and energy homeostasis.^30^ This could lead to immune system dysfunction and altered brain function. In contrast, childhood adversity in the AOMS group was associated with decreased methylation, suggesting that there may be different biological consequences in individuals with MS who experience later disease onset.

Our study has several limitations. The cross-sectional design precludes causal inference, and the relatively small sample size limits statistical power. The cohort was recruited from a single academic MS centre, which may affect generalizability. Nonetheless, the demographic and socioeconomic characteristics of our sample align with previous studies, lending external validity to our findings. Finally, our use of deeply phenotyped participants with rich biological and social data strengthens internal validity.

In summary, this study provides novel evidence that childhood adversity is associated with greater MS symptom burden, subtle increases in neurologic disability, and, most compellingly, distinct epigenetic signatures, particularly among those with paediatric-onset disease. The identification of gene-specific methylation changes—such as those involving *GDF7*—offers a window into how early environmental stressors may shape disease susceptibility and progression through molecular reprogramming of the immune and nervous systems. Future research should consider longitudinal designs to determine the persistence and prognostic significance of these epigenetic changes over time and investigate their role as biomarkers or therapeutic targets. Incorporating epigenetic analyses into our studies of children and adults with childhood histories of adversity could inform strategies to mitigate the long-term impact of childhood adversity in MS and other neurologic disorders.

## Supplementary Material

fcaf512_Supplementary_Data
