# Supplementary material for: Childhood adversity, allostatic load and epigenetic signatures in paediatric and adult-onset multiple sclerosis
Source: Brain Commun. 2026 Jan 3;8(1):fcaf512. doi: 10.1093/braincomms/fcaf512 (PMC12917236; doi:10.1093/braincomms/fcaf512)
Supplement: fcaf512_Supplementary_Data [file fcaf512_supplementary_data.docx]

**Supplementary Material**

**Sociodemographic Features for POMS and AOMS groups.** As shown in Supplementary Table 1, participants with POMS were significantly more likely than AOMS participants to report current public insurance (56.7% vs. 20.0%, p=0.003). A higher proportion of POMS vs. AOMS participants reported an educational level of some college or less, likely related to their younger age at time of enrollment. Similarly, more participants with AOMS reported full time employment.

**Supplementary Table 1.** **Current Sociodemographic Features for POMS and AOMS groups.**

|  | **Pediatric Onset MS (n=30)** | **Adult Onset MS (n=30)** | **p-value** |
| --- | --- | --- | --- |
| **Sociodemographic Features** |  |  |  |
| *Current Insurance*  Public (i.e. Medicaid)  Private | 17 (56.7%)  13 (43.3%) | 8 (26.7%)  22 (73.3%) | ***0.018*** |
| *Patient education*  Some high school  High school or GED  Some college  Associate’s degree or trade school  Bachelor’s degree  Graduate degree  Missing/unknown | 3 (10.0%)  4 (13.3%)  13 (43.3%)  1 (3.3%)  6 (20.0%)  3 (10.0%)  0 (0.0%) | 1 (3.3%)  1 (3.3%)  3 (10.0%)  3 (10.0%)  10 (33.3%)  8 (26.7%)  4 (13.3%) | ***0.022*** |
| *Employment status*  Employed Full time  Employed Part time  Student Full time  Unemployed for less than 6 months  Unemployed for over 6 months  Missing/unknown | 11 (36.7%)  6 (20.0%)  11 (36.7%)  1 (3.3%)  1 (3.3%)  0 (0%) | 22 (73.3%)  2 (6.7%)  0 (0%)  0 (0%)  1 (3.3%)  5 (16.7%) | ***0.002*** |
| *Cigarette Smoking Status*  Current Smoker  Former Smoker  Non-Smoker | 2 (6.7%)  2 (6.7%)  26 (86.7%) | 2 (6.7%)  5 (16.7%)  23 (76.7%) | 0.480 |

**POMS vs AOMS Subgroups.** MS outcome results were compared between the POMS and AOMS subgroups, shown in Supplementary Table 2. SymptoMScreen scores were similar between the MS subgroups with SymptoMScreen scores of 11.13 (7.75) for POMS subgroup and 12.53 (12.00) for the AOMS subgroup, p=0.593. There was only a significant difference seen in bladder control symptoms with an average score of 0.50 (0.82) for POMS and 1.17 (1.53) for AOMS, p=0.04. There was also a significant difference in median EDSS scores between POMS vs. AOMS at 0.00 vs. 1.00, p=0.007.

Allostatic load analyses compared between the POMS and AOMS subgroups are also shown in Supplementary Table 1. Allostatic load biomarker z-scores were similar between the MS groups except total cholesterol was greater in POMS vs AOMS, 0.57 (1.03) vs. -0.02 (0.78), p=0.014. LDL cholesterol was also greater in POMS vs AOMS, 0.59 (1.11) vs. -0.13 (0.71), p=0.004. Overall, POMS vs. AOMS had a trend towards greater allostatic load index at 0.18 (0.43) vs. -0.02 (0.43), p=0.068. Interestingly, our subgroup had smaller BMI than their age- and sex-matched controls in the NHANES cohort as the BMI z-scores were -0.12 (0.59) and -0.26 (0.67) for POMS and AOMS groups, respectively.

Of note, albumin values were excluded from analyses as all participants had outliers compared to their NHANES matched control cohort (53/60 patients with negative z-scores, mean -0.83, SD 0.62, range -2.16 to 0.74), which was thought to represent differences in laboratory testing methods.

**Supplementary Table 2. Outcomes between POMS and AOMS subgroup**

|  | **Pediatric Onset MS (n=30)** | **Adult Onset MS (n=30)** | **p-value** |
| --- | --- | --- | --- |
| **MS Outcomes ^a^** |  |  |  |
| *SymptoMScreen score, mean (SD)*  Walking  Hand Function  Spasticity  Bodily Pain  Sensory symptoms  Bladder control  Fatigue  Vision  Dizziness  Cognitive function  Depression  Anxiety  **SymptoMScreen Composite score** | 0.43 (0.82)  0.67 (0.84)  0.97 (0.85)  0.87 (0.94)  1.00 (1.05)  0.50 (0.82)  1.77 (1.28)  0.70 (0.87)  0.73 (1.11)  0.90 (1.00)  1.03 (1.19)  1.57 (1.59)  11.13 (7.75) | 0.53 (0.94)  0.67 (1.18)  0.90 (1.08)  0.90 (1.16)  1.17 (1.14)  1.17 (1.53)  1.43 (1.38)  1.03 (1.45)  0.73 (1.23)  1.23 (1.41)  1.20 (1.42)  1.63 (1.79)  12.53 (12.00) | 0.661  1.000  0.782  0.903  0.547  ***0.040***  0.336  0.286  1.000  0.294  0.624  0.879  0.593 |
| **EDSS total**, median [IQR] | 0.00  [0.00-0.25] | 1.00  [0.00-2.25] | ***0.007*** |
| **Allostatic Load Variables** |  |  |  |
| *Allostatic Load Biomarker z-scores, mean (SD) ^b,c^*  BMI  MAP  Total Cholesterol  Triglycerides  HDL  LDL  Hgb A1c  HS CRP  Urine albumin creatinine ratio  **Allostatic Load Index, TOTAL** | -0.12 (0.59)  0.50 (0.87)  0.58 (1.03)  0.21 (0.69)  -0.38 (0.79)  0.59 (1.11)  -0.24 (0.96)  0.32 (1.49)  0.16 (1.28)  0.18 (0.43) | -0.26 (0.67)  0.42 (0.85)  -0.02 (0.78)  0.21 (0.98)  -0.44 (0.85)  -0.13 (0.71)  -0.24 (0.61)  0.30 (1.28)  -0.05 (0.24)  -0.02 (0.43) | 0.381  0.699  ***0.014***  0.989  0.760  ***0.004***  0.995  0.952  0.396  0.068 |
| *Legend: Pediatric-onset MS (POMS), Adult-onset MS (AOMS), Body mass index (BMI), High density lipoprotein (HDL), low density lipoprotein (LDL), hemoglobin A1c (Hgb A1c), high sensitivity C-reactive protein (HS-CRP)* | | | |
| ^a^ *Age, disease duration, time reported in mean years (SD) and p-values are reported from independent t-tests. EDSS reported in median [IQR] and p-values are reported from Mann-Whitney U test.* | | | |
| *^b^ BMI was assessed in kg/m2, MAP in mmHg, cholesterol values and CRP in mg/dL, HgbA1c in %.* | | | |
| *^c^ Note that albumin values were excluded from final analyses as all participants had outliers compared to NHANES, which was thought to represent differences in laboratory testing methods.* | | | |

**Participants meeting criteria for childhood adversity vs. not meeting criteria.** Supplementary Table 3 demonstrates the characteristics of individuals meeting criteria for high childhood adversity vs low childhood adversity. There were no significant differences in demographics. Individuals with high childhood adversity had greater SymptoMScreen scores, with participants with low childhood adversity exposure having mean scores of 7.54 (5.928) and participants with high childhood adversity exposure having mean scores of 15.12 (11.305), p=*0.003.* Participants experiencing high childhood adversity also had significantly greater walking, bodily pain, fatigue, vision, cognitive function, and depression scores. There were also trends seen among hand function and dizziness scores. Median EDSS scores were also significantly greater among participants experiencing high childhood adversity, 0.000 [0-0.05] vs. 1.000 [0-2.0], p=0.028. There were no significant differences in allostatic load scores between categories of childhood adversity criteria. EDSS scores were also compared by EDSS of 0 and EDSS ≥1 in Supplementary Table 4.

**Supplementary Table 3. Characteristics of individuals meeting criteria for Childhood Adversity.**

|  | **Childhood adversity, low**  **(n=26)** | **Childhood adversity, high (n=34)** | **p-value** |
| --- | --- | --- | --- |
| **Demographics** |  |  |  |
| *MS Subgroup*  Pediatric Onset MS  Adult Onset MS | 14 (53.8%)  12 (46.2%) | 16 (47.1%)  18 (52.9%) | 0.602 |
| *Sex*  Male  Female | 10 (38.5%)  16 (61.5%) | 13 (38.2%)  21 (61.8%) | 0.986 |
| *Race*  White  Black  American Indian or Alaskan  Asian  Native Hawaiian or Pacific Islander  Other  Unknown | 15 (57.7%)  5 (19.2%)  0 (0.0%)  2 (7.7%)  0 (0.0%)  4 (15.4%)  0 (0.0%) | 15 (44.1%)  11 (32.4%)  0 (0.0%)  1 (2.9%)  0 (0.0%)  4 (11.8%)  3 (8.8%) | 0.331 |
| *Ethnicity*  Hispanic/Latino  Not Hispanic/ Latino  Other | 17 (65.4%)  7 (26.9%)  2 (7.7%) | 20 (58.8%)  12 (35.3%)  2 (5.9%) | 0.778 |
| *Current Insurance*  Public (i.e. Medicaid)  Private | 11 (42.3%)  15 (57.7%) | 12 (35.3%)  22 (64.7%) | 0.580 |
| Age of MS onset | 21.02 (5.98) | 21.21 (5.55) | 0.900 |
| Age at time of enrollment | 26.93 (5.78) | 27.49 (5.78) | 0.711 |
| Disease duration at time of enrollment | 5.92 (3.14) | 6.29 (3.29) | 0.661 |
| Time from MS symptom onset to MS diagnosis | 0.40 (0.98) | 0.75 (1.55) | 0.312 |
| Time from MS symptom onset to first DMT | 0.71 (1.07) | 1.29 (1.73) | 0.116 |
| Time from MS symptom onset to high efficacy DMT | 2.36 (2.70) | 2.98 (3.22) | 0.448 |
| *Ever on high efficacy DMT*  Current/history of high efficacy DMT  Never on high efficacy DMT | 24 (92.3%)  2 (7.7%) | 29 (85.3%)  5 (14.7%) | 0.688 |
| *Current DMT by efficacy*  High efficacy  Moderate efficacy  Missing/unknown | 21 (84.0%)  4 (16.0%)  0 | 28 (82.4%)  6 (17.6%)  0 | 0.868 |
| **MS Outcomes** |  |  |  |
| *SymptoMScreen score, mean (SD)*  Walking  Hand Function  Spasticity  Bodily Pain  Sensory symptoms  Bladder control  Fatigue  Vision  Dizziness  Cognitive function  Depression  Anxiety  **SymptoMScreen score, TOTAL** | 0.15 (0.613)  0.38 (0.637)  0.72 (0.792)  0.54 (0.859)  0.88 (0.971)  0.58 (1.065)  1.04 (1.113)  0.38 (0.637)  0.42 (0.758)  0.65 (1.093)  0.58 (0.987)  1.27 (1.564)  7.54 (5.928) | 0.74 (.963)  0.88 (1.200)  1.09 (1.055)  1.15 (1.105)  1.24 (1.156)  1.03 (1.381)  2.03 (1.337)  1.24 (1.394)  0.97 (1.359)  1.38 (1.231)  1.53 (1.376)  1.85 (1.743)  15.12 (11.305) | ***0.009***  0.060  0.148  ***0.024***  0.218  0.172  ***0.003***  ***0.005***  0.070  ***0.021***  ***0.004***  0.184  ***0.003*** |
| EDSS score, median [IQR] | 0.000  [0-0.05] | 1.000  [0-2.0] | ***0.028*** |
| **Allostatic Load Variables** |  |  |  |
| *Allostatic Load Biomarker z-scores, mean (SD) ^a, b^*  BMI  MAP  Total Cholesterol  Triglycerides  HDL  LDL  Hgb A1c  HS CRP  Urine albumin creatinine ratio  **Allostatic Load Index, TOTAL** | -0.124 (0.806)  0.218 (0.840)  0.250 (0.920)  0.220(0.800)  -0.385 (0.734)  0.210 (0.880)  -0.396 (0.573)  0.157 (0.943)  0.173 (1.327)  0.036 (0.457) | -0.241 (0.460)  0.642(0.836)  0.303 (0.991)  0.203 (0.884)  -0.432 (0.874)  0.240 (1.081)  -0.124 (0.921)  0.431 (1.638)  -0.048 (0.239)  0.111 (0.421) | 0.479  0.057  0.834  0.937  0.828  0.909  0.192  0.451  0.359  0.515 |
| *Legend: Pediatric-onset MS (POMS), Adult-onset MS (AOMS), Body mass index (BMI), High density lipoprotein (HDL), low density lipoprotein (LDL), hemoglobin A1c (Hgb A1c), high sensitivity C-reactive protein (HS-CRP)* | | | |
| 1. *BMI was assessed in kg/m2, MAP in mmHg, cholesterol values and CRP in mg/dL, HgbA1c in %.* | | | |
| 1. *Note that albumin values were excluded from final analyses as all participants had outliers compared to NHANES, which was thought to represent differences in laboratory testing methods.* | | | |

**Supplementary Table 4.** **Childhood Adversity and the relationship to EDSS scores >0.** EDSS scores were compared between individuals with low childhood adversity vs high childhood adversity, first among all participants and then stratified by POMS and AOMS subgroups. Results of Fisher’s exact tests and odds ratios are shown.

| **EDSS by cutoff** | **History of childhood adversity** | **EDSS = 0** | **EDSS ≥ 1** | **p-value** | **Odds Ratio** |
| --- | --- | --- | --- | --- | --- |
| **All participants**  (n=59) | Low | 19 (54%) | 6 (25%) | ***0.034*** | 3.56 |
|  | High | 16 (46%) | 18 (75%) |  |  |
| **POMS**  (n=30) | Low | 14 (61%) | 0 (0%) | ***0.007*** | 22.89^a^ |
|  | High | 9 (39%) | 7 (100%) |  |  |
| **AOMS**  (n=29) | Low | 5 (42%) | 6 (35%) | 1.000 | 1.31 |
|  | High | 7 (58%) | 11 (65%) |  |  |
| Values are n (column percentages). 2-tailed Fisher’s exact tests are reported given small sample sizes of some cells  ^a^ A continuity correction of 0.5 was applied to this table with a zero cell, which should be interpreted cautiously due to sparse data | | | | | |

**Preliminary Epigenetic Analysis.** To map DNA methylation differences between patient subgroups, we performed RRBS and detected ~3.6x10^6^ unique CpG sites (11% of all CpGs in the human genome) after filtering, with coverage >5x in over 80% of samples and median coverage of ~21x per library (ranging from 17-30x). Unsupervised analysis of global CpG methylation levels in individual patients in the entire group did not show any segregation based on MS subgroup or exposure childhood home adversity (Supplementary Figure 1).

ward distance


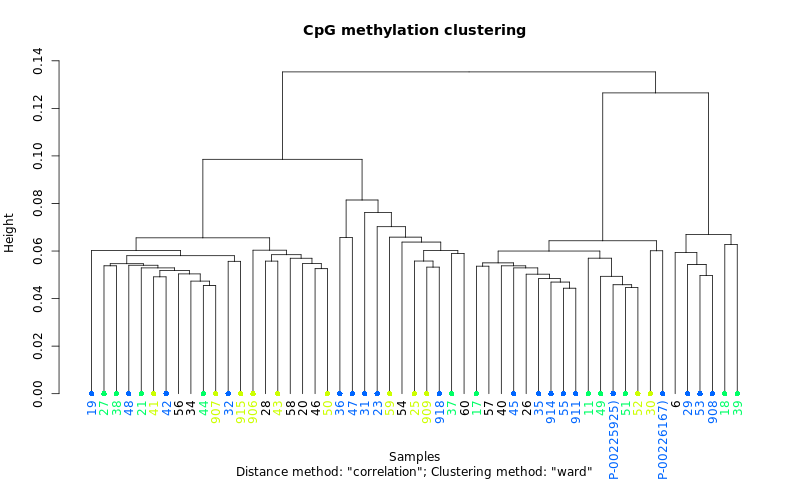

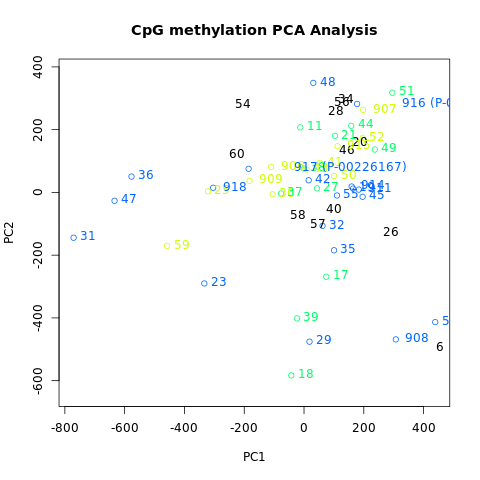


**A**

**B**

**Supplementary Figure 1. (A)** Hierarchical clustering plot of global CpG methylation of individual patients. **(B)** Principal component analysis of CpG methylation. PC1 explains 7% variance, while PC2 5%. In both panels, each dot represents a patient. Patients are stratified using the following color scheme: POMS-low CA, green (n=11); POMS-high CA, black (n=12); AOMS-low CA, yellow (n=11); AOMS-high CA, blue (n=19). *Legend: pediatric-onset MS (POMS), adult-onset MS (AOMS), childhood adversity (CA).*

**Data Availability:**

All RRBS data generated in this study are available at [https://www.ncbi.nlm.nih.gov/geo/](https://urldefense.com/v3/__https:/www.ncbi.nlm.nih.gov/geo/__;!!MXfaZl3l!cD8xN3r8KNz6qyBp-EUftq_iuLC4O-CwHgHrhWYNe09CVQ2meN_QKTfgMJqwtv5_C_G4G19JkvtQvFMjN01EBppuNkncy117ekpp8Q$) under accession number GSE306738. The code used to analyze the data is available at [https://github.com/KianKoh-Lab/Braincom.-2025](https://urldefense.com/v3/__https:/github.com/KianKoh-Lab/Braincom.-2025__;!!MXfaZl3l!cD8xN3r8KNz6qyBp-EUftq_iuLC4O-CwHgHrhWYNe09CVQ2meN_QKTfgMJqwtv5_C_G4G19JkvtQvFMjN01EBppuNkncy10JAlQDiQ$) .
